# *Shigella*-Induced Emergency Granulopoiesis Protects Zebrafish Larvae from Secondary Infection

**DOI:** 10.1128/mBio.00933-18

**Published:** 2018-06-26

**Authors:** Alexandra R. Willis, Vincenzo Torraca, Margarida C. Gomes, Jennifer Shelley, Maria Mazon-Moya, Alain Filloux, Cristina Lo Celso, Serge Mostowy

**Affiliations:** a Section of Microbiology, MRC Centre of Molecular Bacteriology and Infection (CMBI), Imperial College London, London, United Kingdom; b Department of Life Sciences, MRC Centre of Molecular Bacteriology and Infection (CMBI), Imperial College London, London, United Kingdom; c Department of Life Sciences, Imperial College London, London, United Kingdom; d The Francis Crick Institute, London, United Kingdom; e Department of Immunology and Infection, London School of Hygiene and Tropical Medicine, London, United Kingdom; Pasteur Institute

**Keywords:** emergency granulopoiesis, neutrophils, *Shigella*, stem cells, zebrafish

## Abstract

Emergency granulopoiesis is a hematopoietic program of stem cell-driven neutrophil production used to counteract immune cell exhaustion following infection. Shigella flexneri is a Gram-negative enteroinvasive pathogen controlled by neutrophils. In this study, we use a *Shigella*-zebrafish (Danio rerio) infection model to investigate emergency granulopoiesis *in vivo*. We show that stem cell-driven neutrophil production occurs in response to *Shigella* infection and requires macrophage-independent signaling by granulocyte colony-stimulating factor (Gcsf). To test whether emergency granulopoiesis can function beyond homoeostasis to enhance innate immunity, we developed a reinfection assay using zebrafish larvae that have not yet developed an adaptive immune system. Strikingly, larvae primed with a sublethal dose of *Shigella* are protected against a secondary lethal dose of *Shigella* in a type III secretion system (T3SS)-dependent manner. Collectively, these results highlight a new role for emergency granulopoiesis in boosting host defense and demonstrate that zebrafish larvae can be a valuable *in vivo* model to investigate innate immune memory.

## IMPORTANCE

*Shigella* is an important human pathogen of the gut. Emergency granulopoiesis is the enhanced production of neutrophils by hematopoietic stem and progenitor cells (HSPCs) upon infection and is widely considered a homoeostatic mechanism for replacing exhausted leukocytes. In this study, we developed a *Shigella*-zebrafish infection model to investigate stem cell-driven emergency granulopoiesis. We discovered that zebrafish initiate granulopoiesis in response to *Shigella* infection, via macrophage-independent signaling of granulocyte colony-stimulating factor (Gcsf). Strikingly, larvae primed with a sublethal dose of *Shigella* are protected against a secondary lethal dose of *Shigella* in a type III secretion system (T3SS)-dependent manner. Taken together, we show that zebrafish infection can be used to capture *Shigella*-mediated stem cell-driven granulopoiesis and provide a new model system to study stem cell biology *in vivo*. Our results also highlight the potential of manipulating stem cell-driven granulopoiesis to boost innate immunity and combat infectious disease.

## OBSERVATION

Hematopoiesis is the production of cellular blood components. Infection and inflammation can disrupt normal hematopoiesis by exhausting immune cells. Neutrophils are key to control bacterial infection, and increased demand transiently increases neutrophil production by inducing a program of “emergency granulopoiesis” to offset neutrophil loss (1). Emergency granulopoiesis is mediated by the proliferation and differentiation of hematopoietic stem and progenitor cells (HSPCs), which are stimulated directly by pathogen-associated molecular patterns (PAMPs) and indirectly by cytokines (1).

Mouse models have contributed significantly to our understanding of hematopoiesis (2). However, the murine bone marrow presents limited opportunities for directly monitoring hematopoietic dynamics by microscopy. The zebrafish (Danio rerio) is the most widely used nonmammalian vertebrate animal model and a valuable model to study vertebrate hematopoiesis *in vivo* (3). The transparent zebrafish larva enables high-resolution intravital imaging of blood cells in fluorescent transgenic reporter lines and has revealed fundamental aspects of hematopoietic stem cell (HSC) development (4). Zebrafish larvae comprise an innate immune system highly homologous to that of humans (5). Recent studies using zebrafish have described that sterile proinflammatory signaling from myeloid cells can direct HSC development (6–8), and it can be expected that investigation of hematopoiesis in the context of inflammation will continue to provide insights into stem cell biology.

The zebrafish is an important model to study the cell biology of infection *in vivo* (9). A recent study using infection of zebrafish larvae with Salmonella enterica serovar Typhimurium, a Gram-negative bacterial pathogen, has provided mechanistic insight into the signals that mediate emergency granulopoiesis (10). Specifically, a model was proposed in which the macrophage-released cytokine granulocyte colony-stimulating factor (Gcsf) stimulates HSPC proliferation upon infection. However, studies have indicated that the hematopoietic response to infection is pathogen specific, and a more complete understanding of the signaling pathways that govern hematopoiesis is of major therapeutic relevance. The Gram-negative enteroinvasive bacterium Shigella flexneri is an inflammatory paradigm instrumental in the discovery of mechanisms underlying infection control, including nucleotide-binding oligomerization domain (NOD)-like receptors (NLRs), neutrophil extracellular traps (NETs), bacterial autophagy, and interferon-inducible guanylate-binding proteins (GBPs) (11–14). The *Shigella*-zebrafish infection model has previously been used to test novel strategies to fight antimicrobial resistance, and has revealed key roles for bacterial autophagy and the cytoskeleton in host defense and inflammation control (13, 15–17). In this study, we demonstrate that zebrafish larvae initiate stem cell-driven emergency granulopoiesis in response to *Shigella* infection and uncover a fundamental role for emergency granulopoiesis in boosting innate immunity.

### A *Shigella*-zebrafish infection model to study emergency granulopoiesis.

**FIG 1:**
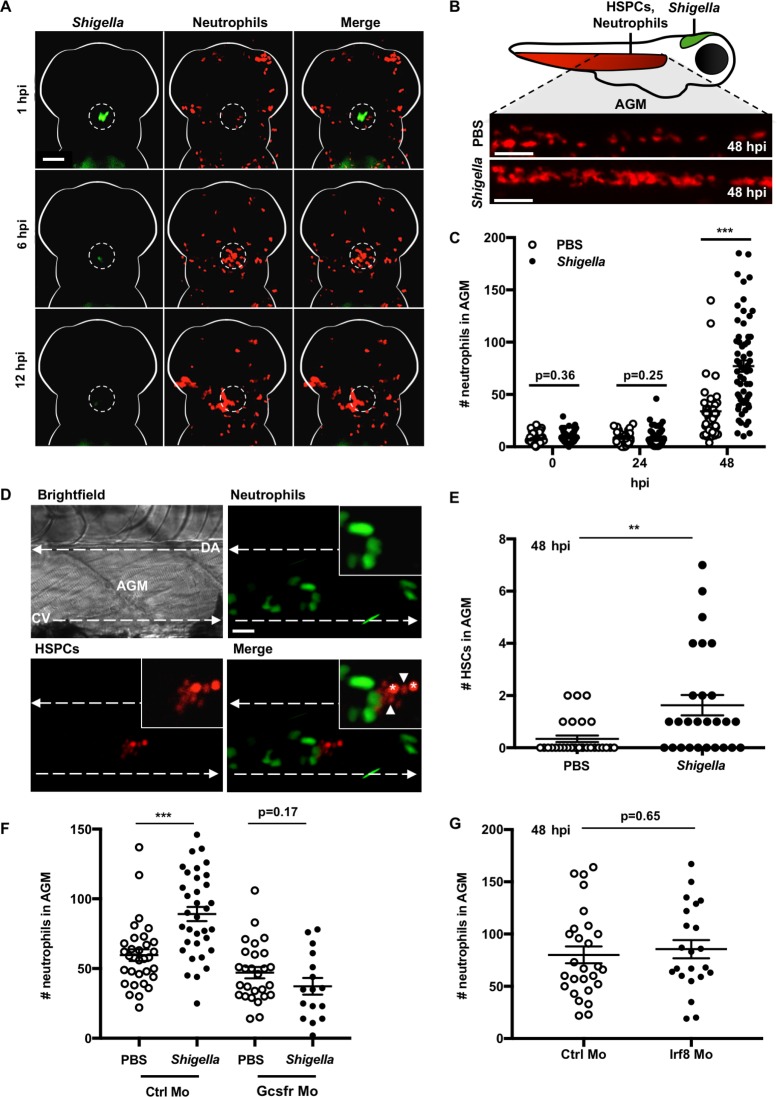
Development of a *Shigella*-zebrafish infection model to study emergency granulopoiesis. (A to C) At 2 dpf, *lyz*::dsRed zebrafish larvae with red neutrophils were injected in the HBV with PBS or a low dose (0.5 × 10^3^ to 2.0 × 10^3^ CFU) of GFP^+^ S. flexneri M90T and imaged by fluorescence stereomicroscopy. (A) Representative images of the infection site in a single larva over time (Movie S1). Scale bars, 100 µm. (B) Schematic of the zebrafish larva, highlighting the AGM region of neutrophil and HSC quantifications. Shown are representative images of the AGM of PBS-injected or *Shigella*-infected larvae at 48 hpi. Scale bars, 100 µm. (C) Quantifications of neutrophils from PBS-injected (open circles) or *Shigella*-infected (closed circles) larvae as in panel B. Circles represent counts from individual larvae. Data were pooled from 4 independent experiments using *n* ≥ 10 larvae per condition per experiment. Means ± SEM are shown (horizontal bars). *P* values between conditions at cognate time points were determined by unpaired two-tailed Student’s *t* test. Signiﬁcance was deﬁned as *P* < 0.05. ***, *P* < 0.001. (D) Tg(*runx*::mCherry)/Tg(*mpx*::GFP) larvae with red HSPCs and green neutrophils were injected at 2 dpf in the HBV with a low dose of S. flexneri M90T, and images of the AGM were captured by confocal microscopy (100× objective). Dashed arrows represent vasculature. DA, dorsal aorta; CV, cardinal vein. Cells expressing high levels of mCherry (*) were considered HSCs; cells expressing lower levels of mCherry were considered HPCs (i.e., HSC progeny [arrowheads]). Maximum-intensity z-projection images are shown. Scale bars, 20 µm. (E) Tg(*runx*::GFP) larvae with green HSCs were injected with a low dose of mCherry^+^ S. flexneri M90T as in panel D. HSCs in the AGM of PBS-injected (open circles) or *Shigella*-infected (closed circles) larvae were quantified at 48 hpi. Circles represent counts from individual larvae. Data were pooled from 3 independent experiments using *n* ≥ 6 larvae per condition per experiment. Means ± SEM are shown (horizontal bars). *P* values between conditions were determined by unpaired two-tailed Student’s *t* test. Signiﬁcance was deﬁned as *P* < 0.05. **, *P* < 0.01. (F) *lyz*::dsRed larvae were treated with control (Ctrl) or Gcsfr morpholino oligonucleotide (Mo). Morphants were injected in the HBV at 2 dpf with PBS (open circles) or a low dose of GFP^+^ S. flexneri M90T (closed circles). Larvae were imaged by fluorescent stereomicroscopy, and neutrophils in the AGM were quantified at 48 h following treatment. Each circle represents a count from an individual larva. Means ± SEM (horizontal bars) are shown. Data were pooled from three independent experiments using *n* ≥ 6 larvae per condition per experiment. *P* values between conditions were determined by unpaired two-tailed Student’s *t* test. Signiﬁcance was deﬁned as *P* < 0.05. ***, *P* < 0.001. (G) *lyz*::dsRed larvae were treated with control or Irf8 morpholino oligonucleotide. Morphants were injected in the HBV at 2 dpf with a low dose of GFP^+^ S. flexneri M90T. Neutrophil quantifications from the AGM of infected larvae are shown. Circles represent counts from individual larvae. Data were pooled from 3 independent experiments using *n* ≥ 4 larvae per condition per experiment. Means ± SEM are shown (horizontal bars). *P* values between conditions were determined by unpaired two-tailed Student’s *t* test. Significance was defined as *P* < 0.05.

As in human development, zebrafish HSCs emerge from the aorta gonad mesonephros (AGM) (18). Zebrafish HSCs are generated from 30 h post-fertilization (hpf) and migrate to the caudal hematopoietic tissue (CHT), where they begin leukocyte production. To study neutrophil production and HSC activity in response to bacterial infection, we developed a *Shigella*-zebrafish infection model. Larvae were injected in the hindbrain ventricle (HBV) at 2 days post-fertilization (dpf) with a low dose (0.5 × 10^3^ to 2.0 × 10^3^ CFU) of S. flexneri M90T. Survival and bacterial burden were monitored over 48 h post-infection (hpi). In this case, infection has no effect on larval mortality (~100% survival) and neutrophils successfully control *Shigella* infection, with the majority of bacteria cleared by 24 hpi (see Fig. S1A and B in the supplemental material). To monitor the interplay between *Shigella* and neutrophils, we performed HBV infections of green fluorescent protein-expressing (GFP^+^) S. flexneri in Tg(*lyz*::dsRed)^*nz50*^ (herein *lyz*::dsRed) transgenic zebrafish larvae, in which neutrophils express dsRed fluorescent protein. Imaging of larvae by fluorescent stereomicroscopy showed neutrophil recruitment to the infection site from 1 hpi and engulfment of *Shigella* alongside an obvious and rapid reduction in GFP^+^ *Shigella* fluorescence (Fig. 1A; see Movie S1 in the supplemental material). Quantifications performed at the whole-animal level show a slight reduction in neutrophil numbers 24 hpi, and local neutrophil depletion by *Shigella* at infection sites can be observed in real time (Fig. S1C). These data are consistent with studies demonstrating that neutrophils are essential to control *Shigella* infection *in vivo* (13) and suggest that a successful host response to *Shigella* infection requires emergency granulopoiesis.

10.1128/mBio.00933-18.1 FIG S1  Development of a *Shigella*-zebrafish infection model to study emergency granulopoiesis. (A and B) At 2 dpf, WT AB zebrafish larvae were injected in the HBV with PBS (open circles) or a low dose (0.5 × 10^3^ to 2.0 × 10^3^ CFU) of GFP^+^ S. flexneri M90T (closed circles). (A) Survival curves were pooled from 3 independent experiments using *n* ≥ 10 larvae per condition per experiment. The *P* value between conditions was determined by log-rank Mantel-Cox test. Signiﬁcance was defined as *P* < 0.05. (B) Enumerations of live bacteria from *Shigella*-infected larvae. Only viable larvae were included in the analyses. Circles represent individual larvae. Data were pooled from 3 independent experiments using up to 3 larvae per time point per experiment. Means ± SEM are shown (horizontal bars). *P* values (versus 0 hpi) were determined by unpaired two-tailed Student’s *t* test. Signiﬁcance with Bonferroni’s correction was deﬁned as *P* < 0.025. ***, *P* < 0.0005. (C) At 2 dpf, *lyz*::dsRed zebrafish larvae with red neutrophils were injected in the HBV with PBS (open circles) or a low dose of GFP^+^ S. flexneri M90T (closed circles). Larvae were imaged by fluorescence stereomicroscopy, and neutrophils in the whole larva were quantified at 24 hpi. Circles represent counts from individual larvae. Data were pooled from 2 independent experiments using *n* ≥ 4 larvae per condition per experiment. Means ± SEM are shown (horizontal bars). *P* values between conditions were determined by unpaired two-tailed Student’s *t* test. Signiﬁcance was deﬁned as *P* < 0.05. **, *P* < 0.01. (D) *lyz*::dsRed zebrafish larvae with red neutrophils were injected systemically in the caudal vein with PBS (open circles) or a low dose of GFP^+^ S. flexneri M90T (closed circles) as in panel C. Larvae were imaged by fluorescence stereomicroscopy, and neutrophils in the AGM were quantified. Circles represent counts from individual larvae. Data were pooled from 2 independent experiments using *n* ≥ 4 larvae per condition per experiment. Means ± SEM are shown (horizontal bars). *P* values between conditions were determined by unpaired two-tailed Student’s *t* test. Signiﬁcance was deﬁned as *P* < 0.05. ***, *P* < 0.001. (E) At 2 dpf, *lyz*::dsRed zebrafish larvae with red neutrophils were injected in the HBV with PBS (open circles) or a low dose of T3SS^−^ S. flexneri M90T (closed circles). Larvae were imaged by fluorescence stereomicroscopy, and neutrophils in the AGM were quantified. Circles represent counts from individual larvae. Data were pooled from 2 independent experiments using *n* ≥ 8 larvae per condition per experiment. Means ± SEM are shown (horizontal bars). *P* values between conditions at cognate time points were determined by unpaired two-tailed Student’s *t* test. Signiﬁcance was deﬁned as *P* < 0.05. *, *P* < 0.05. (F) At 2 dpf, *mpeg1*::*G*/*U*::mCherry larvae with red macrophages were injected in the HBV with PBS or a low dose of GFP^+^ S. flexneri M90T and imaged by fluorescence stereomicroscopy. Shown are quantifications of macrophages from PBS-injected (open circles) or *Shigella*-infected (closed circles) larvae. Circles represent counts from individual larvae. Data were pooled from 3 independent experiments using up to 10 larvae per condition per experiment. Means ± SEM are shown (horizontal bars). *P* values between conditions at cognate time points were determined by unpaired two-tailed Student’s *t* test. Signiﬁcance was deﬁned as *P* < 0.05. *, *P* < 0.05. (G) Standard control morpholino oligonucleotide (Mo)-injected (Ctrl) or Gcsfr morphant WT AB larvae were sacrificed at 2 dpf, and larval homogenates were subjected to RT-PCR to detect alternative splicing following treatment with Gcsfr morpholino oligonucleotide. (H) WT AB larvae were injected with either PBS or a low dose of GFP^+^ S. flexneri M90T in the HBV. RNA was extracted from pools of 5 larvae at 24 hpi, and expression of *gcsfa* mRNA transcripts was determined by RT-qPCR. Means ± SEM (horizontal bars) are shown. Data were pooled from three independent experiments. *P* values (versus PBS controls) were determined by unpaired two-tailed Student’s *t* test. Signiﬁcance was deﬁned as *P* < 0.05. ***, *P* < 0.001. (I) At 2 dpf, control (open circles) and Irf8 (closed circles) morphant *lyz*::dsRed zebrafish larvae with red neutrophils were imaged by fluorescence stereomicroscopy. Leukocyte quantifications from the whole larva are shown. Circles represent counts from individual larvae. Data were pooled from up to 3 independent experiments using up to 8 larvae per condition per experiment. *P* values between conditions were determined by unpaired two-tailed Student’s *t* test. Signiﬁcance was deﬁned as *P* < 0.05. ***, *P* < 0.001. (J) At 2 dpf, untreated (open circles) or metronidazole-treated (closed circles) *mpeg1*::*G*/*U*::mCherry zebrafish larvae were imaged by fluorescence stereomicroscopy. Macrophage quantifications from the whole larva are shown. Circles represent counts from individual larvae. Data were pooled from 3 independent experiments using up to 8 larvae per condition per experiment. Means ± SEM are shown (horizontal bars). *P* values between conditions at cognate time points were determined by unpaired two-tailed Student’s *t* test. Signiﬁcance was deﬁned as *P* < 0.05. ***, *P* < 0.001. (K) At 2 dpf, control (open circles) and metronidazole-treated (closed circles) *mpeg1*::*G*/*U*::mCherry/Tg(*mpx*::GFP)^*i114*^ larvae with green neutrophils were injected in the HBV with a low dose of GFP^+^ S. flexneri M90T. Neutrophil quantifications from the AGM of infected larvae are shown. Circles represent counts from individual larvae. Data were pooled from 3 independent experiments using *n* ≥ 4 larvae per condition per experiment. Means ± SEM are shown (horizontal bars). *P* values between conditions were determined by unpaired two-tailed Student’s *t* test. Signiﬁcance was deﬁned as *P* < 0.05. Download FIG S1, PDF file, 0.3 MB. Copyright © 2018 Willis et al. 2018 Willis et al. This content is distributed under the terms of the Creative Commons Attribution 4.0 International license.

10.1128/mBio.00933-18.3 MOVIE S1  At 2 dpf, *lyz*::dsRed zebrafish larvae with red neutrophils were injected in the HBV with a low dose (0.5 × 10^3^ to 2.0 × 10^3^ CFU) of GFP^+^ S. flexneri M90T and imaged by fluorescence stereomicroscopy. z-stacks were acquired at 5-min intervals from 24 hpi. Download MOVIE S1, AVI file, 2.5 MB. Copyright © 2018 Willis et al. 2018 Willis et al. This content is distributed under the terms of the Creative Commons Attribution 4.0 International license.

To test for emergency granulopoiesis, we injected the HBV of *lyz*::dsRed zebrafish larvae with phosphate-buffered saline (PBS) or a low dose of S. flexneri. To capture production of new neutrophils, we imaged the larval AGM at 24 and 48 hpi (Fig. 1B). Quantifications revealed a significant increase (2.4 ± 0.5-fold) of neutrophils in *Shigella*-infected larvae at 48 hpi (Fig. 1C). A similar increase in neutrophil production is observed following the clearance of systemic *Shigella* administered via caudal vein injection (Fig. S1D). *Shigella* virulence is dependent upon the bacteria’s type III secretion system (T3SS) (19). To determine the role of the T3SS in the induction of emergency granulopoiesis, we performed HBV infections of zebrafish larvae using a T3SS-deficient (T3SS^−^) strain of S. flexneri (Δ*mxiD*). In agreement with a role for *Shigella* virulence in the induction of emergency granulopoiesis, neutrophil production is only weakly induced (1.5 ± 0.2-fold) by T3SS^−^ infection (Fig. S1E). To test for a global increase in myeloid cell production following infection, Tg(*mpeg1*::Gal4-FF)^*gl25*^/Tg(*UAS-E1b*::*nfsB*.mCherry)^*c264*^ (herein *mpeg1*::*G*/*U*::mCherry) larvae were infected with *Shigella* in the HBV, and mCherry-expressing (mCherry^+^) macrophages were quantified. Macrophage counts in the AGM are not significantly affected by *Shigella* infection (Fig. S1F). Taken together, these results show that infection by *Shigella* drives a neutrophil-specific hematopoietic response and that *Shigella* infection of zebrafish can be used to study factors underlying emergency granulopoiesis *in vivo*.

### Macrophage-independent signaling of Gcsf mediates stem cell-driven granulopoiesis during *Shigella* infection.

HSCs are increasingly recognized as important reactive components to infection (20). Runx1 is a transcription factor crucial for HSC development (21). To visualize stem cell-driven granulopoiesis, we outcrossed Tg(*runx*::mCherry)^*cz2010*^ zebrafish that have mCherry-expressing HSCs with Tg(*mpx*::GFP)^*114*^ zebrafish that have GFP-expressing neutrophils. Double-transgenic larvae were infected with *Shigella* and imaged by high-resolution confocal microscopy. Here, photostability of the mCherry protein confers a dim fluorescence onto the stem cell’s immediate progeny. During *Shigella* infection, we observed that multiple high-mCherry-expressing HSCs are surrounded by low-mCherry progenitor cells and proximal to progenitor cells are GFP-positive neutrophils (Fig. 1D). We therefore hypothesized that HSC proliferation and differentiation underlie neutrophil production in response to *Shigella* infection. To test for increased stem cell proliferation upon *Shigella* infection, we quantified HSCs in the AGM of Tg(*runx*::eGFP)^*cz2009*^ larvae. Consistent with results obtained using *Salmonella* infection of zebrafish (10), we observed significantly more HSCs (5.3 ± 1.8-fold) in *Shigella*-infected larvae at 48 hpi compared to uninfected larvae (Fig. 1E). These results indicate that the emergency granulopoietic response to *Shigella* infection is stem cell driven.

Previous work using zebrafish infection suggested that macrophage-derived Gcsf is required for emergency granulopoiesis (10). To test this, we used a morpholino oligonucleotide to deplete the Gcsf receptor (Gcsfr). Consistent with a role for Gcsf in *Shigella*-induced emergency granulopoiesis, Gcsfr morphants showed no significant increase in neutrophil production in the AGM upon infection (Fig. 1F; Fig. S1G). In support of a role for Gcsf in mediating granulopoiesis, quantitative reverse transcription-PCR (RT-qPCR) revealed significant increases in Gcsf expression in *Shigella*-infected larvae at 24 hpi (Fig. S1H). To test the role of macrophages in *Shigella*-induced emergency granulopoiesis, we used a morpholino oligonucleotide to target the myeloid lineage commitment factor Irf8 and deplete macrophages. Surprisingly, Irf8 morphants undergo robust emergency granulopoiesis during *Shigella* infection (Fig. 1G and Fig. S1I). Similar results are obtained using transgenic *mpeg1*::*G*/*U*::mCherry/Tg(*mpx*::eGFP)^*114*^ larvae, in which macrophages were ablated pharmacologically by metronidazole treatment (Fig. S1J and K). Together, these results indicate that macrophage-independent signaling of Gcsf is required for stem cell-driven granulopoiesis during *Shigella* infection.

### *Shigella*-induced emergency granulopoiesis mediates long-term host defense.

**FIG 2:**
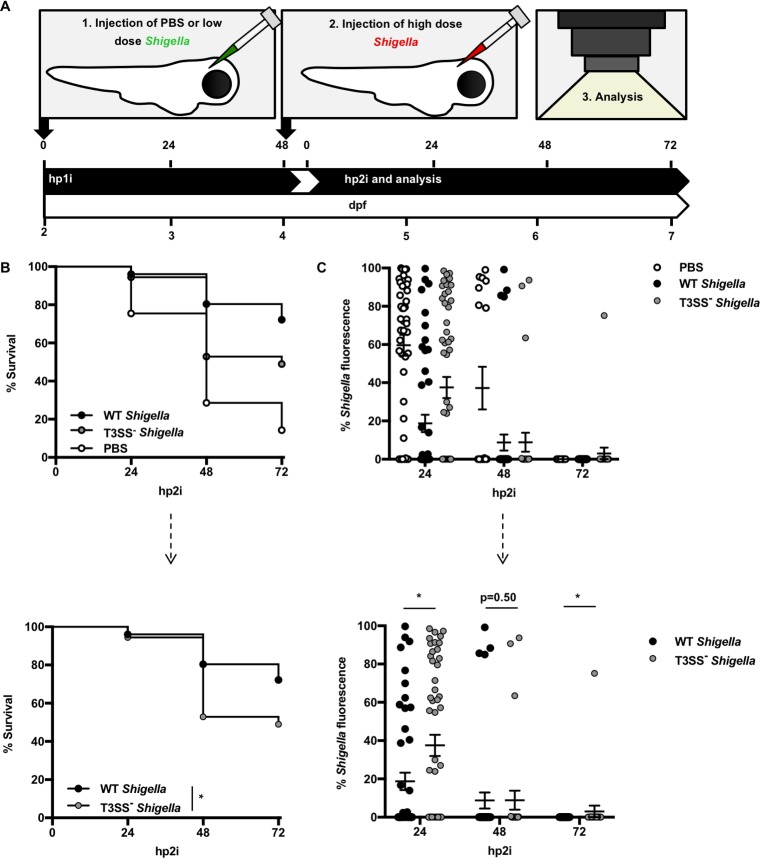
Emergency granulopoiesis mediates long-term host defense. (A) Schematic of reinfection assays. At 2 dpf, wild-type (WT) AB zebrafish larvae were injected in the HBV with PBS or a low dose (0.5 × 10^3^ to 2.0 × 10^3^ CFU) of GFP^+^ S. flexneri M90T. At 4 dpf, i.e., 48 h post-primary injection (hp1i), all larvae were injected with a high dose (>2.0 × 10^4^ CFU) of mCherry-expressing (mCherry^+^) S. flexneri M90T. Analyses were performed on larvae up to 72 h post-secondary infection (hp2i). (B and C) WT AB larvae were injected with PBS (open circles) or “primed” with wild-type or T3SS^−^ GFP^+^ S. flexneri M90T (closed circles), prior to a high dose of mCherry^+^ S. flexneri M90T at 48 hpi, as described above. (B) Survival curves pooled from 4 independent experiments using *n* ≥ 9 larvae per condition per experiment. Up to three larvae per condition were taken for CFU at the 24 and 48 h time points. The top graph represents collated data. The bottom graph represents only *Shigella*-primed larvae, a subset of the above data. The *P* value between conditions was determined by log-rank Mantel-Cox test. Signiﬁcance was deﬁned as *P* < 0.05 (*). (C) Fluorescent mCherry^+^ S. flexneri M90T burden of larvae was imaged by stereomicroscopy over time, and images were analyzed to produce fluorescence intensity measurements (as in Fig. S2A). Data were pooled from 4 independent experiments with *n* ≥ 4 per time point per condition per experiment. The top graph represents collated data. The bottom graph represents only *Shigella*-primed larvae, a subset of the above data. *P* values between conditions at cognate time points were determined by unpaired two-tailed Student’s *t* test. Significance was defined as *P* < 0.05 (*).

Emergency granulopoiesis is widely considered to be a transient homoeostatic mechanism for replacing exhausted leukocytes (1). To test if emergency granulopoiesis can also enhance innate immunity, we developed a *Shigella* reinfection assay (Fig. 2A). For this, larvae at 2 dpf were injected in the HBV with PBS or primed with a low dose (0.5 × 10^3^ to 2.0 × 10^3^ CFU) of GFP^+^ *Shigella*. At 48 hpi, control (naive) or infected larvae were reinfected with a lethal dose (>2.0 × 10^4^ CFU) of mCherry^+^ *Shigella*. Strikingly, priming of larvae with a low dose of *Shigella* rescued ~70% of animals that would have otherwise succumbed to secondary infection and significantly reduced bacterial burden compared to naive larvae (Fig. 2B and C; see Fig. S2A in the supplemental material). At the point of secondary infection (i.e., 0 h post-secondary infection [hp2i]), total neutrophil numbers are similar between naive and primed larvae, indicating that increased protection is not because of increased neutrophil numbers (Fig. S2B). To test the role of the T3SS in triggering protection against reinfection, we primed zebrafish larvae with wild-type or T3SS^−^ *Shigella*. Consistent with inducing only a mild granulopoietic response, T3SS^−^ *Shigella* provides some protection against secondary infection and rescues ~40% of lethal infections; however, the most robust protection is observed following infection with virulent *Shigella* (Fig. 2B and C). Collectively, these results show that emergency granulopoiesis is not solely a homoeostatic mechanism to counteract neutrophil cell death, but can also enhance innate immunity to secondary infection.

10.1128/mBio.00933-18.2 FIG S2  Emergency granulopoiesis mediates long-term host defense. (A) At 2 dpf, WT AB zebrafish larvae were injected in the HBV with PBS or a low dose (0.5 × 10^3^ to 2.0 × 10^3^ CFU) of GFP^+^ S. flexneri M90T. At 4 dpf (i.e., 48 h post-primary injection [hp1i]), all larvae were injected with a high dose (>5.0 × 10^3^ CFU) of mCherry^+^ S. flexneri M90T. Images are representative of the mCherry^+^ S. flexneri burden in the HBV of naive or primed larvae after 24 h following secondary infection. Infection burden was determined by fluorescence stereomicroscopy and is expressed as the percentage of fluorescence of the larval hindbrain (e.g., 42.2% and 1.5%). (B) At 2 dpf, *lyz*::dsRed zebrafish larvae with red neutrophils were injected in the HBV with PBS (closed circles) or a low dose of GFP^+^ S. flexneri M90T (open circles). Larvae were imaged by fluorescence stereomicroscopy, and neutrophils in the whole larva were quantified at 48 hpi. Circles represent counts from individual larvae. Data were pooled from 2 independent experiments using *n* ≥ 4 larvae per condition per experiment. Means ± SEM are shown (horizontal bars). *P* values between conditions were determined by unpaired two-tailed Student’s *t* test. Signiﬁcance was deﬁned as *P* < 0.05. Download FIG S2, TIF file, 0.9 MB. Copyright © 2018 Willis et al. 2018 Willis et al. This content is distributed under the terms of the Creative Commons Attribution 4.0 International license.

### Conclusion.

The transparent zebrafish larva provides a unique system in which to visualize hematopoiesis, infection, and innate immunity *in vivo* (9, 17). In this study, we used *Shigella* infection of zebrafish to investigate stem cell-driven emergency granulopoiesis *in vivo* and discovered that emergency granulopoiesis can be used to boost host defense.

*Shigella* is an inflammatory pathogen controlled by neutrophils (13). In agreement with previous findings using *S*. Typhimurium infection of zebrafish (10), we observe robust neutrophil production in response to S. flexneri infection. Recent studies have shown that HSCs can respond to inflammatory cues and hematopoietic stress (20). Consistent with this, we show that HSCs proliferate and differentiate in *Shigella*-infected larvae. The pathogen-sensing cell types required to produce granulopoietic cytokines and stimulate neutrophil production are poorly understood, although monocytes are suggested to be predominant signaling effectors (1, 10). However, macrophage depletion does not impact emergency granulopoiesis during *Shigella* infection of zebrafish. Recent studies in mice have indicated that epithelial cells are the primary source of granulocyte colony-stimulating factor in response to local Escherichia coli infection (22), and this may also be the case during *Shigella* infection. Considering that infection models for E. coli, *S*. Typhimurium, and S. flexneri are already established (10, 13, 23), the zebrafish is highly suited to address the precise mechanisms underlying the hematopoietic response to these different bacterial pathogens.

The zebrafish does not develop adaptive immunity until ~30 dpf; therefore, larvae provide a unique opportunity to study innate immunity in isolation (5). The nascent field of innate immune memory describes how innate immune cells can exhibit adaptive immune characteristics and enhance host resistance to secondary infection (24). To our knowledge, no studies have tested the ability of emergency granulopoiesis to boost innate immunity. Although *Salmonella* has previously been used to induce granulopoiesis in zebrafish (11), *Shigella* infection of zebrafish provides an exceptional model system to investigate the consequence of emergency granulopoiesis upon secondary infection. We therefore tested a role for emergency granulopoiesis in enhancing host defense using *Shigella* reinfection assays. Remarkably, priming of larvae with *Shigella* reduced pathogen burden and rescued larval survival in response to a secondary infection. *Shigella* infection of zebrafish also can be used to investigate the role of bacterial virulence factors in boosting immune defense. Here, we discovered that a T3SS-deficient strain could induce some emergency granulopoiesis and host protection, although maximum protection against secondary infections is only achieved using virulent, wild-type *Shigella*. In future studies, it will be interesting to determine whether enhanced immunity in primed larvae is an example of inflammatory memory, a phenomenon that has recently been described for skin epithelial stem cells (25), or innate immune memory, a phenomenon that has recently been described for HSCs primed with Mycobacterium bovis bacillus Calmette-Guerin (BCG) (26). Such memory may enable the HSPC compartment to react more efficiently to infection by producing neutrophils with enhanced microbicidal capacity. In a follow-up study, this could be tested by assessment of neutrophils following primary versus secondary infection. It will also be interesting to test the specificity of these responses: for example, by dissecting whether the conferred protection is restricted to S. flexneri or is general against a variety of bacterial pathogens.

In conclusion, we developed a *Shigella*-zebrafish infection model to study hematopoiesis *in vivo*. Using this model, we discovered that emergency granulopoiesis can function beyond homoeostasis and boost innate immunity. An in-depth understanding of the mechanisms governing HSC biology will be important for the therapeutic manipulation of innate immunity in humans and the treatment of hematological disease.

### Ethics statement.

Animal experiments were approved by the Home Office (project licenses PPL 70/7446 and PPL P84A89400) and performed in accordance with the Animals (Scientific Procedures) Act 1986.

### Zebrafish husbandry.

Wild-type AB zebrafish were purchased from the Zebrafish International Resource Center (Eugene, OR). The transgenic lines Tg(*lyz*::dsRed)^*nz50*^, Tg(*mpx*::GFP)^*i114*^, Tg(*runx*::mCherry)^*cz2010*^, Tg(*runx*::eGFP)^*cz2009*^, and Tg(*mpeg1*::Gal4-FF)^*gl25*^/Tg(*UAS-E1b*::*nfsB*.mCherry)^*c264*^ were previously published (27–30). Embryos were obtained from naturally spawning zebrafish, and both control and infected larvae were maintained at 28.5°C in embryo medium (0.5× E2 medium supplemented with 0.3 µg/ml methylene blue) (31). Larvae were staged according to Kimmel et al. (32). For injections and live microscopy, larvae were anesthetized with 200 µg/ml tricaine (Sigma-Aldrich) in embryo medium.

### Zebrafish infection.

Bacterial strains used include wild-type invasive S. flexneri serotype 5a M90T expressing GFP or mCherry protein and a T3SS^−^ noninvasive Δ*mxiD* variant expressing mCherry (33). *Shigella* cells were cultured overnight in Trypticase soy broth (TSB), diluted 50× in fresh TSB, and grown at 37°C until they reached an *A*_600_ of 0.6. Bacteria were harvested by centrifugation, washed, and resuspended in PBS to achieve the desired concentration. For infection, larvae were microinjected in the hindbrain ventricle (HBV) or caudal vein at 2 dpf with up to 1 nl of PBS or a low dose (0.5 × 10^3^ to 2.0 × 10^3^ CFU) of S. flexneri. For reinfection assays, larvae at 2 dpf were injected with either PBS or a low dose of GFP^+^ S. flexneri in the HBV; larvae were confirmed to have cleared GFP^+^ *Shigella* infection (as determined by fluorescence stereomicroscopy) and were infected at 4 dpf (i.e., 48 hpi) with a high dose (>2.0 × 10^4^ CFU) of mCherry^+^ S. flexneri. Injection protocols are as described previously (34). Larvae were maintained in individual wells of a 12-well culture dish for assessment.

### Survival assays.

Larvae were imaged using a light stereomicroscope at time points following infection. Larvae failing to produce a heartbeat or larvae in which bacteria had compromised the hindbrain were considered nonviable.

### Measurement of inocula and bacterial burden.

For enumeration of live bacteria by CFU plating, larvae were mechanically homogenized in lysis buffer (0.4% Triton X-100, PBS). Homogenates were serially diluted and plated on lysogeny broth (LB) agar supplemented with 50 µg/ml carbenicillin. Fluorescent colonies were scored after incubation of plates for 24 h at 37°C. For reinfection assays, S. flexneri burden was determined by fluorescence stereomicroscopy. Here, the larval hindbrain was defined as a “region of interest” and was subjected to thresholding to give the percentage of fluorescence of the hindbrain. Only viable larvae were used for CFU and image fluorescence analysis.

### Morpholino oligonucleotide injection.

Antisense morpholino oligonucleotides were purchased from GeneTools (http://www.gene-tools.com). *gcsfr* (ENSDARG00000045959) was targeted using published morpholino sequence 5′ ATTCAAGCACATACTCACTTCCATT 3′ to block mRNA splicing (10). Macrophages were depleted by targeting *irf8* (ENSDARG00000056407) using published morpholino sequence 5′ AATGTTTCGCTTACTTTGAAAATGG 3′ to block mRNA splicing (35). To control for nonspecific effects, a standard morpholino oligonucleotide with no known target in the zebrafish genome was used (13). Morpholino oligonucleotide solutions were diluted to the desired concentration (1 mM) in 0.1% phenol red solution (Sigma-Aldrich), and 0.8 nl was microinjected into the yolk sack at the 1- to 2-cell stage.

### Microscopy and image analysis.

For *in vivo* time-lapse imaging, larvae were immobilized in 1.5% low-melting-point agarose as previously described (34). Stereomicroscopy was performed using a Leica M205FA microscope and 10× (NA 0.5) dry objective. For Movie S1, z-stacks were acquired every 15 min. For high-resolution confocal microscopy, larvae were positioned in 35-mm-diameter glass-bottom MatTek dishes and imaging was performed using a Zeiss LSM 710 and 10×, 20×, and 40× oil or 63 × oil immersion objectives. Image files were processed using ImageJ/FIJI software (36). Leukocyte and HSC quantifications were performed manually from images taken by stereomicroscopy.

### RT-qPCR.

RNA was extracted from 10 snap-frozen larvae with an RNeasy minikit (Qiagen) and reverse-transcribed using a QuantiTect reverse transcription kit (Qiagen) as per the manufacturer’s instructions. Template cDNA was subjected to PCR using primers for *gcsf* (ENSDARG00000102211), as previously described (10). Quantitative PCR (qPCR) was performed on a Rotor-GeneQ thermocycler (Qiagen) and samples run in technical duplicate with SYBR green master mix (Applied Biosystems). Primers against housekeeping gene *ef1a1l1* (13) and the threshold cycle (2^−ΔΔ*CT*^) method (37) were used to normalize cDNA.

### Validation of morpholino oligonucleotide depletion.

For validation of alternative Gcsfr splicing, RNA was isolated from a pool of control and Gsfr morphants at 2 dpf. cDNA was prepared as described above in “RT-qPCR” and used as a template for RT-PCR using OneTaq DNA polymerase (New England Biolabs) and primers Gcsfr-FW (5′ CATCCGTCTCGCTTGTGCTT 3′) and Gcsfr-Rv (5′ GGTGGGACCGCATAAACCTT 3′).

### Drug treatments.

To deplete macrophages, *mpeg1*::*G*/*U*::mCherry larvae were treated with 10 mM metronidazole (Sigma-Aldrich) in embryo medium supplemented with 1% dimethyl sulfoxide (DMSO) (Sigma-Aldrich) from 24 hpf, as previously described (28).

### Statistical analyses.

Statistical tests were performed using Prism software (GraphPad Software, Inc.). Statistical significance of survival curves was determined using the log rank Mantel-Cox test. In all other cases, statistical significance was determined using unpaired two-tailed Student’s *t* test; analyses were performed on raw values for cell counts, log_10_ values for CFU counts, and log_2_ values for gene expression data. Bonferroni’s posttest was applied in cases of multiple testing, as specified in the figure legends. Data are represented as mean ± standard errors of the mean (SEM).
