# Supplementary material for: Shigella-Induced Emergency Granulopoiesis Protects Zebrafish Larvae from Secondary Infection
Source: mBio. 2018 Jun 26;9(3):e00933-18. doi: 10.1128/mBio.00933-18 (PMC6020294; doi:10.1128/mBio.00933-18)

Figure S1. Development of a *Shigella*-zebrafish infection model to study emergency granulopoiesis

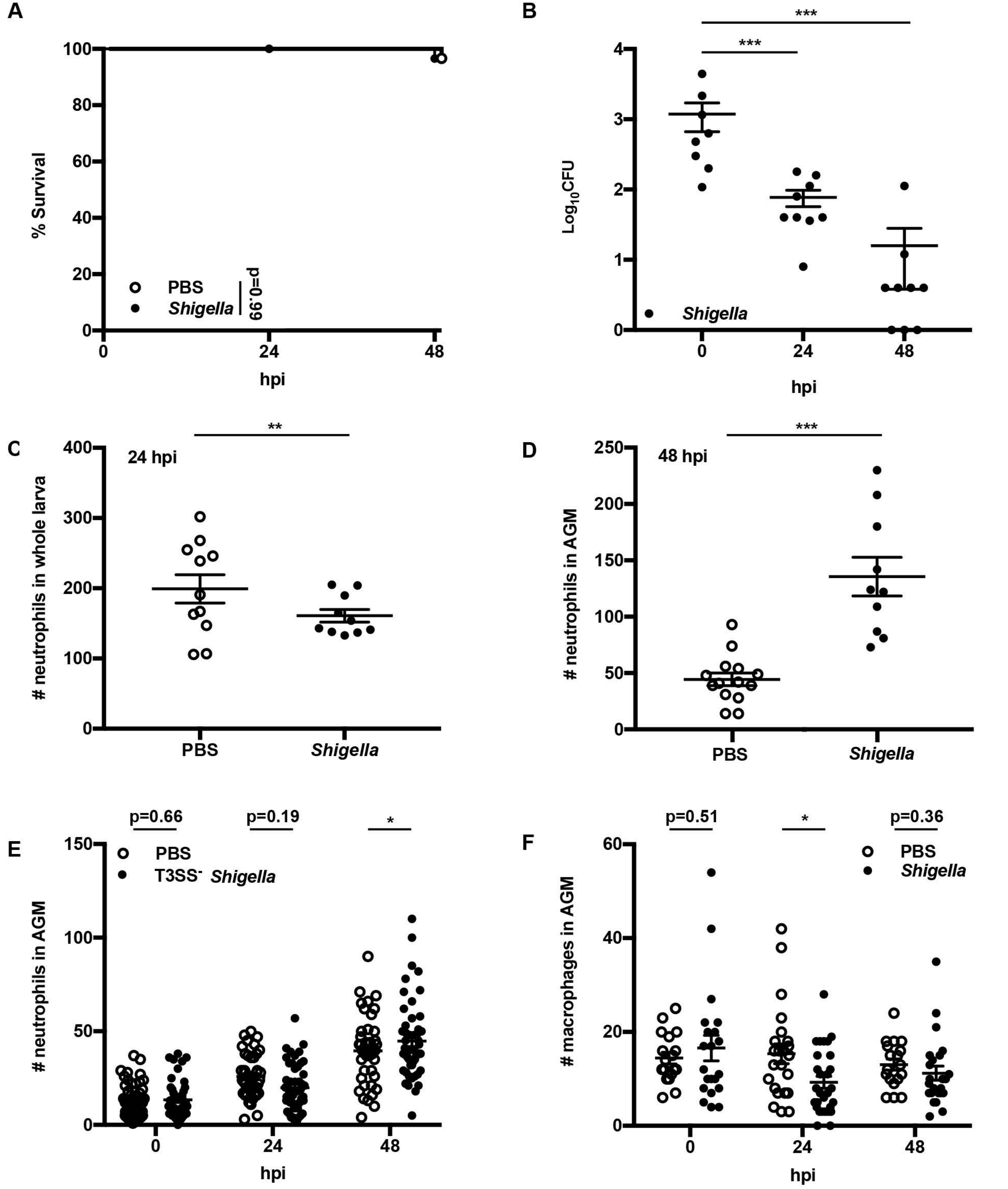

Figure S1. Development of a *Shigella*-zebrafish infection model to study emergency granulopoiesis

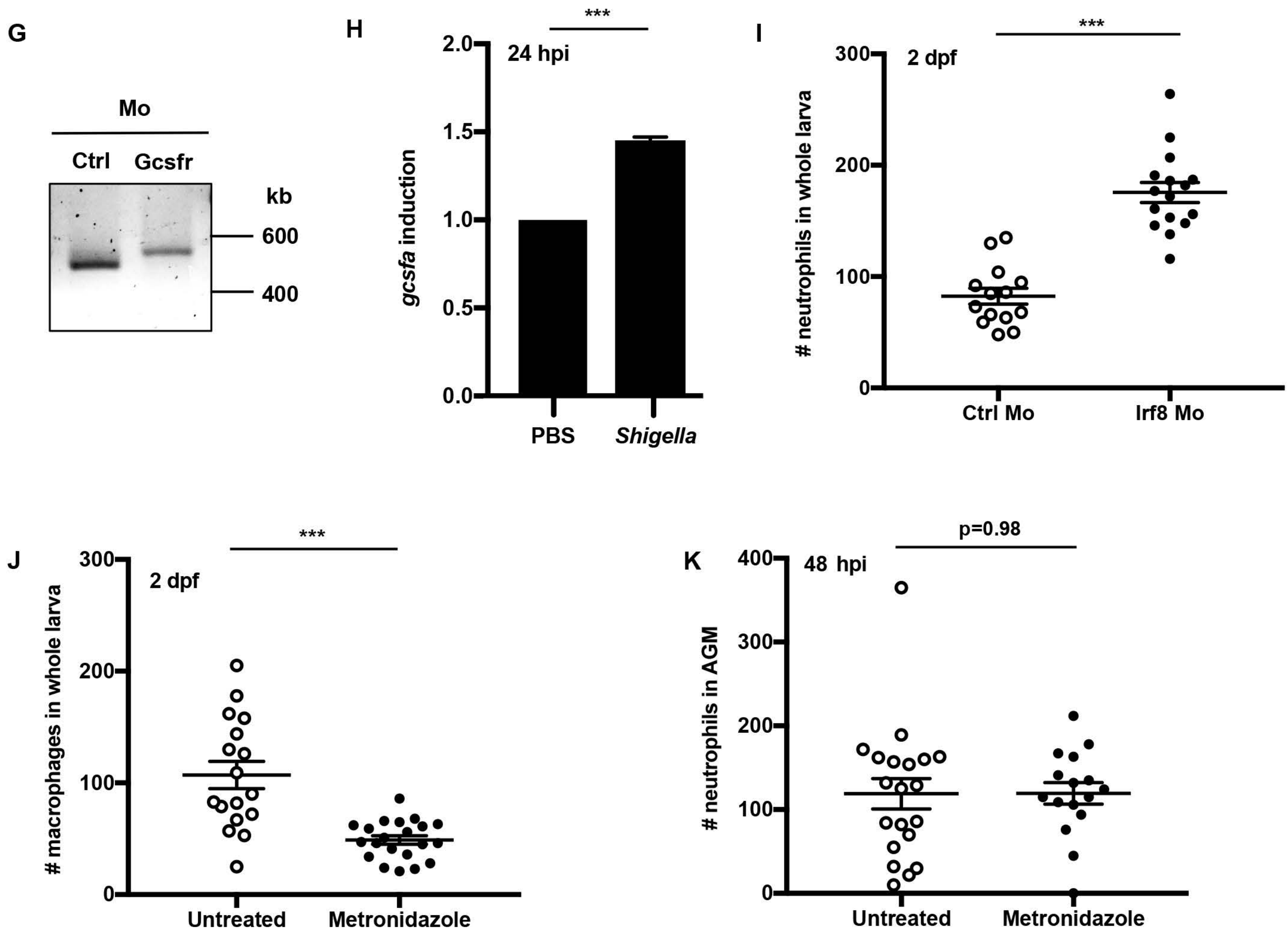

Supplement: FIG S1 [file mbo003183949sf1.pdf]
