# Supplementary figures and images for: Shigella-Induced Emergency Granulopoiesis Protects Zebrafish Larvae from Secondary Infection
Source: mBio. 2018 Jun 26;9(3):e00933-18. doi: 10.1128/mBio.00933-18 (PMC6020294; doi:10.1128/mBio.00933-18)

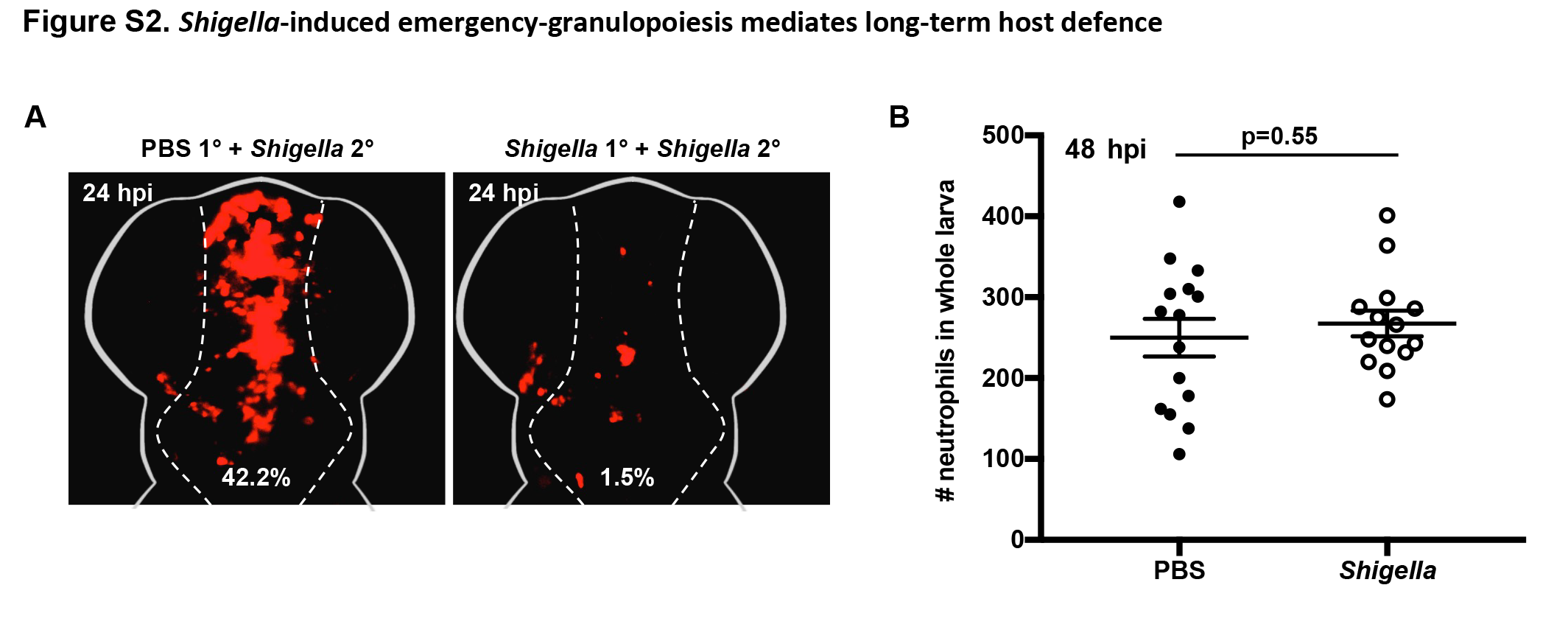

Supplement: FIG S2 [file mbo003183949sf2.tif]
